# NMDA receptor C-terminal signaling in development, plasticity, and disease

**DOI:** 10.12688/f1000research.19925.1

**Published:** 2019-08-30

**Authors:** Giles E Hardingham

**Affiliations:** 1 UK Dementia Research Institute, University of Edinburgh, Edinburgh, EH16 4TJ, UK; 2 Centre for Discovery Brain Sciences, Edinburgh Medical School, University of Edinburgh, Edinburgh, EH8 9XD, UK

**Keywords:** Excitotoxicity, Neurodegeneration, NMDA Receptor, Calcium, Development, Plasticity

## Abstract

The NMDA subtype of ionotropic glutamate receptor is a sophisticated integrator and transducer of information. NMDAR-mediated signals control diverse processes across the life course, including synaptogenesis and synaptic plasticity, as well as contribute to excitotoxic processes in neurological disorders. At the basic biophysical level, the NMDAR is a coincidence detector, requiring the co-presence of agonist, co-agonist, and membrane depolarization in order to open. However, the NMDAR is not merely a conduit for ions to flow through; it is linked on the cytoplasmic side to a large network of signaling and scaffolding proteins, primarily via the C-terminal domain of NMDAR GluN2 subunits. These physical interactions help to organize the signaling cascades downstream of NMDAR activation. Notably, the NMDAR does not come in a single form: the subunit composition of the NMDAR, particularly the GluN2 subunit subtype (GluN2A–D), influences the biophysical properties of the channel. Moreover, a growing number of studies have illuminated the extent to which GluN2 C-terminal interactions vary according to GluN2 subtype and how this impacts on the processes that NMDAR activity controls. We will review recent advances, controversies, and outstanding questions in this active area of research.

## Introduction

NMDARs (N-methyl-D-aspartate [NMDA] receptors) are glutamate-gated cation-passing channels that play a major role in the CNS ^1, 2^. They are permeant to Ca ^2+^, which mediates many of the consequences of NMDAR activity, including synaptic modification, activity-dependent development, and neuroprotective signaling. Moreover, inappropriate NMDAR activity contributes to neurotoxicity and synaptotoxicity in a variety of acute and chronic pathological situations ^3– 5^. Most NMDARs contain two obligate GluN1 subunits, plus two GluN2 subunits, of which there are four types (2A–D), with GluN2A and GluN2B predominant in the forebrain, GluN2C prevalent in the cerebellum, and GluN2D found mainly in the midbrain. The GluN2 subtype dictates many biophysical properties of the NMDAR, including agonist affinity, open probability, and deactivation kinetics, and so influences synaptic NMDAR-evoked currents and downstream signaling ^2^. During forebrain development, there is a shift in the subunit composition of NMDARs, from near-exclusively GluN2B-containing NMDARs to populations of NMDARs containing GluN2A representation––both GluN1 _2_–GluN2A _2_ diheteromeric receptors as well as GluN1 _2_–GluN2A–GluN2B triheteromeric receptors ^2^.

NMDARs do not exist in isolation on the plasma membrane, and so the consequences of their activation do not merely depend on the profile of ionic flux upon their activation. The cytoplasmic C-terminal domains (CTDs) of NMDAR subunits are linked to a signaling complex––a network of signaling and scaffolding molecules ^6^. In the case of GluN2 subunits, the CTDs are very large in vertebrate animals––around 600 amino acids (GluN2A and 2B) or 400 amino acids (GluN2C and 2D) compared to around 100 amino acids in invertebrate orthologs ^7^. As will be discussed in this review, CTD-associated proteins influence the events downstream of NMDAR activation, and differential association with different NMDAR subunits contributes to the functional diversity of NMDAR signaling. There will be an emphasis on the cellular and molecular consequences of NMDAR activity rather than behavioral outputs. Moreover, while a small number of studies have probed the role of protein interactions with GluN2C and GluN2D CTDs in receptor trafficking and degradation ^8– 11^, the overwhelming majority are centered on the CTDs of GluN2A and 2B, which will be the focus of this review.

## NMDAR interactions with cytoplasmic proteins depend on subunit composition

Members of the membrane-associated guanylate kinase (MAGUK) family of scaffold proteins, including post-synaptic density (PSD) protein 95 (PSD-95), PSD-93, and SAP102, were the first proteins to be identified as associated with NMDAR subunit CTDs ^12^, but comprehensive proteomic studies quickly identified many more ^13^. The existence of NMDAR signaling complexes recruited by the subunit CTDs also has implications for NMDAR signaling diversity. The large CTDs of GluN2A and GluN2B have diverged during evolution ^7^, raising the possibility that the CTD subtype influences the composition of the NMDAR signaling complex and downstream consequences of NMDAR activity. An early difference identified was a high-affinity binding site for CaMKII present on the GluN2B CTD (CTD ^2B^) but absent on the GluN2A CTD (CTD ^2A^) ^14^. More recently, the generation of knock-in mice with targeted alterations to CTD ^2B^ and CTD ^2A^ has enabled a more comprehensive analysis of the roles of specific CTDs in endogenous NMDAR complex assembly. For example, the GluN2A ^2B(CTR)^ mouse was created that expresses GluN2A with its CTD replaced with that of GluN2B, while the GluN2B ^2A(CTR)^ mouse has the reciprocal swap ^15^. Thus, regardless of the GluN2A:2B ratio in forebrain neurons, the CTDs are all of the 2B type in GluN2A ^2B(CTR)^ mice or the 2A type in GluN2B ^2A(CTR)^ mice. From analysis of these mice, it was found that there was a preferential association of MAGUK proteins with CTD ^2B^ over CTD ^2A^ ^15, 16^. However, isolation of native NMDAR supercomplexes (protein complexes associated with the NMDAR channel complex itself ^17^) revealed something more fundamental ^18^. These supercomplexes were found to exist in two broad size ranges, 0.85 MDa and 1.5M Da, and, by analyzing complexes from wild-type, GluN2A ^2B(CTR)^, and GluN2B ^2A(CTR)^ mice, the authors found that CTD ^2B^ was essential for the recruitment of the large 1.5 MDa complex (or group of complexes) containing a number of scaffolding and signaling proteins, suggesting that components of the 1.5 MDa complex may play a role in CTD ^2B^-specific signaling. Also of note, there is likely to be a degree of heterogeneity in these complexes, as the cumulative mass of all the proteins identified in each complex exceeds the size of them in both cases ^18^, and families of MAGUK supercomplexes have recently been identified ^19^. Moreover, there is evidence that GluN2 CTDs are intrinsically disordered but may undergo conformational switching in response to signals, influencing their interactions with other proteins, providing further scope for diversity in complexes ^7, 20, 21^. Thus, it is clear that the divergence of GluN2 CTDs through evolution has caused a corresponding divergence in their protein-binding partners.

NMDAR mobility and surface dynamics play important roles in regulating synaptic NMDAR currents and plasticity and are composition sensitive ^22, 23^. The recruitment of the NMDAR into the large 1.5 MDa supramolecular complex by CTD ^2B^ ^18^ raises the question of how this affects mobility. Paradoxically, quantum dot-based studies in cultured hippocampal neurons have indicated that GluN2B-containing NMDARs are more mobile than GluN2A-containing NMDARs and have a lower dwell time at the synapse ^24^. However, there is significant overlap between the distributions of membrane diffusion rate of 2A- and 2B-containing NMDARs, which is not surprising, since many endogenous hippocampal NMDARs are triheteromeric, containing one each of GluN2A and GluN2B ^25, 26^. Given the range of diffusion rates observed in 2B-containing NMDARs, it remains a possibility that those NMDARs with CTD ^2B^-recruited supramolecular complexes are a subpopulation of low-mobility 2B-containing NMDARs. Note, however, that NMDAR mobility is controlled by interactions with extracellular matrix proteins ^23, 27^, so cytoplasmic protein interactions are not the only determinant.

## The role of GluN2 CTD subtypes in forebrain synaptogenesis

The first few weeks of rodent forebrain development are characterized by a burst of synaptogenesis, as well as a gradual incorporation of GluN2A into previously GluN2B-dominated NMDARs. This led to hypotheses that the changing NMDAR subunit composition may play a role in regulating synaptogenesis. Indeed, it was shown that CTD ^2B^ was important for hippocampal excitatory synaptogenesis and that excessive GluN2A CTDs impaired synaptogenesis (measured by mEPSC frequency ^28^). These conclusions were based on over-expression of wild-type and chimeric subunits based on GluN2A and GluN2B with their CTDs reciprocally exchanged ^28^. Moreover, the critical domain was identified as the GluN2B CTD-specific CaMKII interaction site ^28^. Furthermore, a recent study employing hippocampal slice cultures reported that GluN2B’s CaMKIIα interaction site is important for correct basal AMPA receptor-mediated synaptic transmission ^29^, based on viral/CRISPR deletion of the endogenous GluN2B gene in the culture and ectopic expression of mutant versus wild-type subunits. In contrast, studies on hippocampal slices from knock-in GluN2B ^2A(CTR)^ and GluN2A ^2B(CTR)^ mice revealed normal synaptogenesis, AMPA receptor currents, and mEPSC frequency ^15^, suggesting that the development of these aspects of synaptic physiology are not sensitive to GluN2 CTD subtype. Moreover, two independently generated knock-in mice harboring a mutation specifically in the GluN2B CTD CaMKII interaction site (hereafter GluN2B ^∆CaMKII^ mice) were also found to have normal hippocampal excitatory synaptogenesis ^30, 31^. These differences in observations between ectopic expression of mutant subunits and knock-in mouse mutants may reflect over-expressed subunits being trafficked and signaling differently to endogenously expressed subunits. For example, one difference between over-expression of GluN2B mutants and knock-in models is that the former may have the effect of altering the relative stoichiometry of GluN2B and CaMKIIα, which could influence CaMKIIα interactions in the PSD. Quantitative mass spectrometry has shown that in the PSD, CaMKIIα is 30–50 times more abundant than NMDARs ^32, 33^. Thus, altering the CaMKIIα interaction domain of GluN2B in a knock-in model may not strongly influence the interactions that the majority of CaMKIIα molecules have within the PSD. However, over-expression of GluN2B could more strongly perturb total synaptic CaMKIIα interactions and consequently have a stronger physiological effect. Another possibility is that a germline mutation in place since the very beginning of development causes other adaptive processes to compensate for the absence of the GluN2B–CaMKIIα interaction domain.

In contrast to the numerous studies on the role of GluN2 CTDs in synaptogenesis, their role in another aspect of neuronal development, dendritic outgrowth and arborization, is less studied, despite the involvement of synaptic NMDAR activity in this process ^34^. Interestingly, a recent study employing transgenic mice over-expressing the GluN2A ^2B(CTR)^ and GluN2B ^2A(CTR)^ alleles found that GluN2A ^2B(CTR)^ over-expression, but not GluN2B ^2A(CTR)^ over-expression, drove enhanced CA1 dendritic outgrowth without affecting branching or other morphological indices ^35^. It will be of interest in the future to link the potential role of CTD ^2B^ in promoting dendritic outgrowth and its ability to organize large signaling supercomplexes ^18^.

## The role of GluN2 CTD subtypes in controlling NMDAR subunit composition

NMDAR subunit composition strongly influences the kinetics of ion flux ^1, 2^, with important functional consequences. For example, in the visual cortex, there is a developmental and experience-dependent incorporation of GluN2A into synaptic NMDARs. The resulting increase in 2A:2B ratio is believed to be causally linked to shifts in the stimulus threshold for frequency-dependent transition between LTD and LTP ^36^, so-called meta-plasticity. The basis for this is the slower deactivation kinetics of GluN2B-containing NMDARs leading to a greater capacity for signal integration at low frequencies and thus a lower minimum frequency for the induction of LTP.

Given the functional importance of forebrain NMDAR composition, the mechanism by which GluN2A becomes incorporated into NMDARs at the expense of GluN2B during development is of importance. One recent model proposes that the switch is driven by a series of phosphorylation events centered on the CTD of GluN2B (CTD ^2B^), initiated by CaMKIIα binding to its 2B-specific interaction site ^37– 39^. CaMKIIα has been proposed to recruit casein kinase 2 (CK2) to the GluN2B CTD, which in turn phosphorylates CTD ^2B^ at serine-1480, leading to dissociation of the MAGUK–Fyn complex and reduction in CTD ^2B^ tyrosine-1472 phosphorylation ^38, 39^. It has been suggested that these events destabilize GluN2B’s presence at the synapse and ultimately trigger endocytosis via AP-2-mediated endocytosis ^38– 40^. However, this sequence of events was arrived at following *in vitro* experiments involving the ectopic over-expression of mutant subunits. Thus, the role of this pathway in the developmental switch of endogenous NMDAR subunits both *in vitro* and *in vivo* remained unclear. Analysis of the developmental change in 2A:2B ratio in neurons from GluN2B ^∆CaMKII^ and GluN2A ^2B(CTR)^ knock-in mice *in vitro* and *in vivo* showed that the GluN2B–CaMKIIα interaction site was not needed for the developmental shift in 2A:2B ratio and indeed proceeded normally when both GluN2A and GluN2B possessed identical CTDs (the GluN2A ^2B[CTR]^ mouse) ^31^.

However, the developmental shift in NMDAR subunit composition is thought to have two components: one an intrinsic process, and one regulated in a bidirectional manner by sensory experience ^41, 42^. Since CK2 inhibition blocks the acute activity-dependent increase in the 2A:2B ratio in hippocampal slices ^39^, it is possible that the experience-dependent regulation of NMDAR subunit composition *in vivo* (exemplified by studies in the visual cortex) does indeed require the GluN2B CaMKII site and the CaMKIIα–CK2 signaling axis. The dynamic and specific removal of GluN2A from the synapse in the visual cortex after sensory deprivation, and its delivery back to the synapse upon sensory stimulation ^36^, suggests that there must be some way that GluN2 subunits are differentially recognized by the activity-dependent machinery responsible for changing the 2A:2B ratio. Study of experience-dependent changes in NMDAR composition in the visual cortex of GluN2A ^2B(CTR)^, GluN2B ^2A(CTR)^, and GluN2B ^∆CaMKII^ knock-in mice will illuminate the roles of CTD subtype-specific sequences in this process. It should be noted, though, that other determinants of the differential activity dependency of GluN2A and GluN2B insertion have been proposed. In particular, ectopic expression of a range of 2A/2B chimeric subunits identified a GluN2B-specific putative N-glycosylation site in the extracellular loop between M3 and M4 transmembrane domains as being necessary for enabling activity-independent insertion of GluN2B, with the corresponding sequence in GluN2A conferring an activity dependency on insertion ^43^. However, given differences in results obtained by ectopic expression of mutant subunits, compared to the corresponding mutant knock-in mouse, demonstration of the importance of this site would benefit from its germline mutation.

## GluN2 C-terminal domains and synaptic plasticity

NMDAR activation mediates several forms of synaptic plasticity, both potentiation and depression, as well as homeostatic forms ^44– 46^. Shortly after the distinct GluN2 subunits were identified, there began conjecture as to whether specific subunits mediate specific types of potentiation or depression ^1, 2^. Studies have primarily focused on GluN2A-deficient mice and the use of pharmacological tools specific for 2B- or 2A-containing NMDARs ^1, 2^. While the specificity of the 2A-preferring NMDAR antagonists has been questioned ^47^, recent ones are more effective ^48^. A role for a specific GluN2 subtype in a plasticity paradigm can conceivably be due to the particular biophysical properties of those channels (e.g. with regard to frequency-dependent signal integration) or even simply that a specific subunit is predominant at a synapse at the developmental stage under study. However, another alternative is that GluN2 subtype-specific CTD interactions and signaling are important for specific types of plasticity. Helpfully, the GluN2 CTD subtype does not influence the channel gating properties of the NMDAR ^49, 50^, so CTD manipulations can be made without impacting on ionic flux.

An early study using slice cultures employed biolistic siRNA knock-down of endogenous GluN2B, followed by over-expression of siRNA-resistant forms of wild-type GluN2B, GluN2B with its CTD replaced by that of GluN2A (i.e. the GluN2B ^2A[CTR]^ protein), and GluN2A with its CTD replaced by that of GluN2B (i.e. the GluN2A ^2B[CTR]^ protein). The study concluded that CTD ^2B^ was essential for LTP and that CTD ^2A^ potentially even inhibited LTP ^51^. However, analysis of more physiological systems painted a far less dramatic picture. Studies on hippocampal slices from GluN2B ^2A(CTR)^ and GluN2A ^2B(CTR)^ knock-in mice suggest that there is no absolute requirement for a specific CTD for LTP induction but that their relative importance may depend on the stimulation paradigm employed ^15^. For example, NMDAR-dependent theta burst (TBS)-induced LTP of CA3–CA1 connections is normal in GluN2A ^2B(CTR)^ mice, compared to wild-type ^15^, and was modestly (but significantly) enhanced in GluN2B ^2A(CTR)^ mice. Thus, despite the importance of CTD ^2B^ in organizing supramolecular complexes ^17^, TBS-LTP was not compromised. In contrast, GluN2B ^2A(CTR)^ mice did show a deficit in LTP induced by theta-pulse stimulation ^15^, suggesting that the roles of CTDs may be activity pattern dependent. Note that even a synaptic NMDAR with a disrupted local supramolecular complex (e.g. in GluN2B ^2A[CTR]^ mice) is still embedded within the wider network of signaling and scaffolding proteins which make up the PSD. Given this, the signaling machinery that the NMDAR ordinarily recruits directly may still be functionally accessible within the wider PSD. In separate studies, transgenic mice over-expressing the GluN2A ^2B(CTR)^ and GluN2B ^2A(CTR)^ alleles both exhibited enhanced LTP ^52^, a slightly different observation to the studies on the equivalent knock-in mice that may be due to the altered NMDAR expression levels in the transgenic lines.

In the context of CTD roles in synaptic plasticity, the role of the GluN2B-specific high-affinity CaMKII site has been a particular focus of research. In an early study, ectopic expression of GluN2B with its CaMKII site mutated was found to abolish LTP ^53^. Study of a GluN2B ^∆CaMKII^ knock-in mouse harboring mutations in the site (L1298A/R1300Q) revealed a more modest LTP deficit ^30^, even though the authors confirmed that the potentiation was completely CaMKII dependent regardless of genotype. Other sources of CaMKII signaling may be due to CaMKIIα association with GluN1 within the NMDAR ^54^ or activation of the large quantity of CaMKIIα elsewhere in the PSD ^32, 33^. In contrast, an LTP deficit was not observed in a different GluN2B ^∆CaMKII^ knock-in mouse line harboring a L1298A/R1300Q/S1303D mutation ^31^, consistent with studies showing that the entire CTD ^2B^ can be dispensed with and LTP not inhibited ^15^. Aside from the additional mutation at serine-1303 (designed to maximize disruption to the CaMKII site ^14^), the strategy and experimental paradigms of the two studies were similar and the basis for the differing results unclear. Nevertheless, neither study supports the notion of complete dependence on the site as previously thought ^53^. To conclude, while GluN2 CTD subtypes can influence synaptic plasticity, it is likely to be both activity pattern and potentially even pathway dependent. Moreover, much remains to be uncovered regarding the domains and interactions involved.

## GluN2 C-terminal domains, excitotoxicity, and neurodegeneration

During the 1980s, it was established that excessive Ca ^2+^ influx through NMDARs is a major mediator of excitotoxic neuronal death induced by glutamate exposure and contributes to excitotoxic disorders including stroke and traumatic brain injury ^55, 56^. Soon after, it was observed that Ca ^2+^ influx specifically through the NMDAR was more effective at promoting neuronal death than influx through other routes ^57, 58^, implicating a functional or physical coupling of the NMDAR to a Ca ^2+^-responsive effector of neuronal death. One such effector is neuronal nitric oxide synthase (nNOS), physically tethered to GluN2 subunits via a bridging scaffold protein (PSD-95), which interacts with both nNOS and the extreme C-terminal PDZ ligand of GluN2 subunit CTDs ^59^. nNOS is a Ca ^2+^-dependent enzyme which, if overactivated, contributes to NMDAR-dependent excitotoxicity ^60, 61^. A cell-permeable peptide mimetic of the GluN2B PSD-95 interaction domain (a PDZ ligand) designed to reduce NMDAR–nNOS coupling via PSD-95 is neuroprotective in stroke models in rodents and monkeys ^62, 63^ and successfully completed a phase II trial for safety and efficacy for iatrogenic micro-strokes during cerebral aneurysm repair ^64^. This peptide, latterly named NA-1, is in clinical trials for stroke ^65^.

While stroke trials for conventional NMDAR antagonists uniformly failed ^66^, there is reason to be more hopeful in the case of NA-1. Physiological synaptic NMDAR activity is essential for brain function and cognition, so antagonists are poorly tolerated ^66^. Moreover, physiological synaptic NMDAR activity promotes a variety of protective effects ^67– 71^, and so targeting the NMDAR downstream of the channel may avoid translational issues that beset stroke trials with conventional NMDAR antagonists ^72^. Conventional antagonists also may suppress protective reconditioning-type responses in marginal brain areas after stroke, such as the induction of antioxidant responses ^72– 74^. Notably, there is evidence that NA-1 neither interferes with NMDAR channel function nor inhibits the pro-survival pathways that are triggered by physiological patterns of synaptic NMDAR activity ^62, 75^. As a caveat, however, others have reported a reduction in surface expression of GluN2B after NA-1 treatment, an effect also observed with positively charged poly-arginine peptides ^76^.

Regardless, the signaling pathways that lead to NMDAR-dependent excitotoxic neuronal death are numerous, and not all rely on the GluN2B–PSD-95–nNOS pathway ^77, 78^. In a study designed to assess the relative roles of CTD ^2B^ versus CTD ^2A^, it was observed that forebrain neurons in the GluN2B ^2A(CTR)^ mouse were resistant to NMDAR-dependent excitotoxic injury *in vitro* and *in vivo* ^16^. However, not all of the protective effects of replacing CTD ^2B^ with CTD ^2A^ were due to a reduction in nNOS activation, pointing to other mechanisms or key domains involved in the pro-death effects of CTD ^2B^. One possibility considered was whether the GluN2 CTD subtype influenced the localization (synaptic versus extrasynaptic) of NMDARs, since extrasynaptic NMDARs couple preferentially to pro-death signaling cascades and pro-death gene expression ^3, 5, 79, 80^. However, no influence of GluN2 CTD subtype on synaptic versus extrasynaptic location was observed ^16^.

In further searching for CTD ^2B^ determinants of pro-death signaling, Vieira *et al*. ^81^ took GluN2B-deficient neurons and ectopically expressed GluN2B subunits with modified C-termini ^81^ and studied vulnerability to excitotoxic oxygen–glucose deprivation. In agreement with the previous study ^16^, the authors found that expressing GluN2B with its C-terminus replaced by that of GluN2A reduced NMDAR-dependent excitotoxicity and also confirmed a role for the GluN2B PDZ ligand. However, they also found that introducing a CaMKII-binding site double mutation (R1300Q/S1303D) reduced toxicity, implicating this domain as a contributor to pro-death NMDAR signaling ^81^. Interestingly, the CTD ^2B^ CaMKII site was also implicated in a separate study ^82^ but as a site that recruited Dapk1 rather than CaMKIIα. The authors proposed that, in response to excitotoxic insults, Dapk1 causes serine-1303 phosphorylation on CTD ^2B^, increasing extrasynaptic NMDAR currents ^82^. Consistent with this, *Dapk1* ^–/–^ neurons were reported to be resistant to excitotoxicity, and a cell-permeable peptide mimetic of the CTD ^2B^ region around serine-1303 disrupted serine-1303 phosphorylation and was neuroprotective ^82^. However, a recent study failed to observe any protection *in vitro* or *in vivo* in an independently generated *Dapk1* ^–/–^ mouse, and the peptide mimetic was found to directly antagonize the NMDAR by virtue of its high positive charge ^83^, casting doubt on CTD ^2B^–Dapk1 signaling being involved in excitotoxicity. Further studies in rodent or human systems ^84^ may resolve this controversy.

Other signaling pathways involved in excitotoxic signaling, aside from NO production, include NADPH oxidase activation, oxidative stress, calpain activation, and mitochondrial Ca ^2+^ overload ^3, 5, 85– 89^. Might any of these be preferentially activated by signaling reliant on CTB ^2B^? Mitochondrial Ca ^2+^ overload via the mitochondrial calcium uniporter (Mcu) is a major contributor to excitotoxicity ^90^. Moreover, imaging data have shown that NMDAR-dependent Ca ^2+^ influx is preferentially coupled to mitochondrial uptake and depolarization compared to other Ca ^2+^ entry routes ^91, 92^. However, the molecular basis for this is unknown. Interestingly, the 1.5 MDa supramolecular complex recruited by CTD ^2B^ ^17^ contains several mitochondrial proteins, including outer mitochondrial membrane proteins VDAC1–3 and VDAC-associated inner mitochondrial proteins ANT1 and ANT2. VDACs allow cytoplasmic Ca ^2+^ to flow into the mitochondrial intermembrane space, which is taken up into the matrix via Mcu. The presence of VDAC and ANT proteins is suggestive of a physical link between certain NMDARs and mitochondria (potentially via CTD ^2B^) which may facilitate Ca ^2+^ transfer.

Another area of interest in the context of pathological GluN2 CTD signaling is in chronic neurodegenerative disease, particularly Alzheimer’s disease (AD). In the AD brain, circumstances can conspire to elevate the level of ambient glutamate, which may be due to a combination of factors, including bioenergetic deficits impairing glutamate homeostasis, inflammatory cells and astrocytes releasing glutamate, as well as reduced astrocytic glutamate transporter expression ^93– 95^. This can lead to a chronic, low-level form of excitotoxicity, progressively impairing synaptic integrity and contributing to neuronal death ^94^. Tonic NMDAR activity acting on extrasynaptic NMDARs also exacerbates the situation by promoting amyloidogenic APP processing ^96, 97^. As such, NMDAR activity is thought to be a mediator of synapse loss induced by amyloid-β, though not by direct binding to the NMDAR ^94, 98^.

While the role for CTD ^2B^ in promoting NMDAR excitotoxicity suggests that it may contribute to any neurological disorder where glutamate homeostasis is disrupted, there is also some evidence that it may act as a specific point of integration for tau and amyloid-β neurotoxicity. A study showed that tau, known to be required for amyloid-β neurotoxicity ^99^, is required for dendritic recruitment of the tyrosine kinase Fyn, which phosphorylates CTD ^2B^ on tyrosine-1472, enhancing CTD ^2B^ association with PSD-95 and potentiating the CTD ^2B^–PSD-95–nNOS neurotoxic cascade ^100^. A separate study implicated amyloid-β–prion protein interactions, rather than tau, in Fyn activation and CTD ^2B^ phosphorylation ^101^. Regardless, a direct testing of the role for CTD ^2B^ in amyloidopathy-associated synapse loss could potentially be provided by crossing AD models onto the GluN2B ^2A(CTR)^ line to determine whether CTD ^2B^ is a potential therapeutic target for AD.

## Concluding remarks

Studies are beginning to tease apart the roles of the NMDAR CTDs in organizing signaling complexes and mediating the downstream effects of NMDAR activation, though many questions remain. An emerging area of NMDAR research is the potential of an activated receptor to signal in an ion flux-independent, metabotropic way (reviewed thoroughly elsewhere ^102^). The role of specific CTD sequences in sensing NMDAR activation and moving in an ion flux-independent manner to alter interactions with proteins ^102, 103^ awaits further investigation. Moreover, the previously under-appreciated diversity in synapse morphology and composition ^104^ suggests comparable diversity in NMDAR CTD-recruited signaling complexes, the functional consequences of which will require study. Moreover, owing to the sheer size of GluN2 CTDs, it is likely that further functionally important domains will be discovered in the coming years.
